# Cytokines and Chemokines as Emerging Biomarkers and Therapeutic Targets in Colorectal Cancer—Narrative Review

**DOI:** 10.3390/ijms27041996

**Published:** 2026-02-19

**Authors:** Weronika Sokólska, Monika Gudowska-Sawczuk, Karolina Orywal

**Affiliations:** Department of Biochemical Diagnostics, Faculty of Pharmacy with the Division of Laboratory Medicine, Medical University of Bialystok, Waszyngtona 15A, 15-269 Bialystok, Poland; weronika.sokolska@sd.umb.edu.pl (W.S.); monika.gudowska-sawczuk@umb.edu.pl (M.G.-S.)

**Keywords:** colorectal cancer, cytokines, chemokines, tumor microenvironment, biomarkers, immunotherapy, CXCL12–CXCR4, IL-2, IL-12

## Abstract

Colorectal cancer (CRC) is a significant global health challenge, characterized by an increasing incidence rate and high mortality rate. Early detection and effective treatment are crucial to improving patients’ quality of life. Cytokines and chemokines are key modulators of the tumor microenvironment, influencing the recruitment of immune cells, angiogenesis, proliferation, and metastasis. This narrative review summarizes the current knowledge regarding the potential diagnostic and therapeutic applications of selected cytokines and chemokines in CRC. We discuss their potential as biomarkers for early detection, prognosis, and prediction of treatment response. We also highlight emerging therapeutic strategies targeting cytokine and chemokine pathways, including immune checkpoint inhibitors, modulation of chemokine signaling, and the direct use of cytokines to enhance antitumor immunity, with particular emphasis on interleukin-6 (IL-6), C-X-C motif chemokine ligand 8 (CXCL8), C-C motif chemokine ligand 2 (CCL2), and the C-X-C motif chemokine ligand 12 (CXCL12)–C-X-C chemokine receptor type 4 (CXCR4) axis, which show consistent associations with tumor stage, metastasis, and treatment response. Integrating cytokine- and chemokine-based approaches with combination therapies could lead to more effective conventional treatments. In summary, this review emphasizes the potential of cytokines and chemokines as diagnostic tools and therapeutic targets, paving the way for more personalized and effective treatment strategies for colorectal cancer.

## 1. Introduction

Colorectal cancer (CRC) is the third most frequently diagnosed cancer and the second leading cause of cancer mortality in both men and women. The incidence of this type of cancer is increasing, and experts expect the incidence of CRC to increase by up to 60% by 2030, which will pose a significant challenge and a greater burden on healthcare systems worldwide [1]. CRC is a latent disease entity that often occurs in an advanced stage. It is characterized by a slow, multi-stage process that takes approximately 5–10 years from the appearance of precancerous lesions to the development of colorectal cancer [2]. Most cases develop from benign precancerous polyp lesions following the somatic inactivation of the tumor suppressor *Adenomatous Polyposis Coli* (*APC*) and progress through a series of genetic and epigenetic changes that accumulate over years or decades, involving mutations in *Kirsten rat sarcoma virus oncogene homologue* (KRAS)/*v-raf murine sarcoma viral oncogene homolog B1* (BRAF), *SMAD family member 4* (SMAD)/*transforming growth factor beta receptor 2* (TGFBR2), *tumor protein p53* (p53), and *phosphatidylinositol-4,5-bisphosphate 3-kinase catalytic subunit alpha* (PIK3CA) [3].

**Figure 1:**
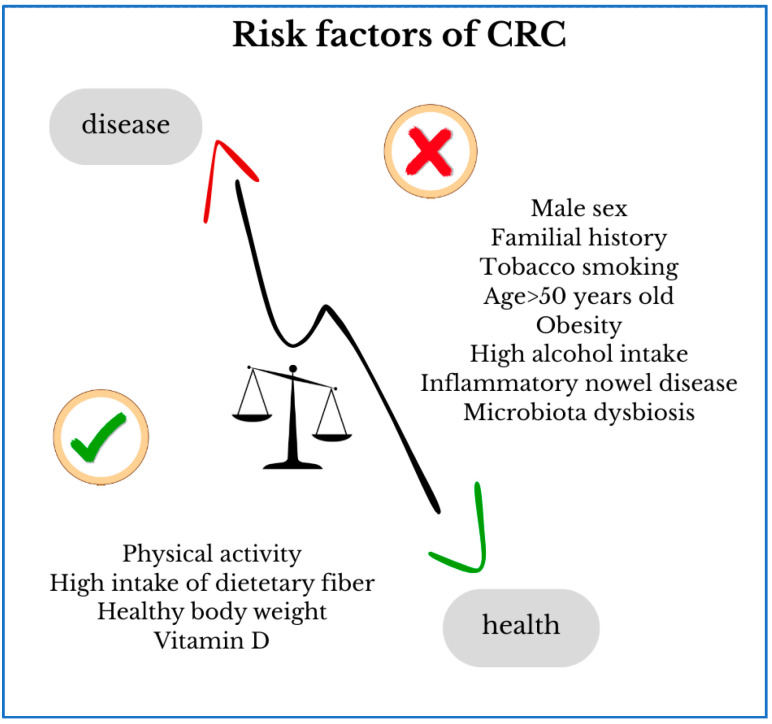
Risk factors of colorectal cancer.

Many factors, including patient-related factors and familial, genetic, and environmental predispositions, have a significant impact on the development of colorectal cancer (Figure 1). The incidence of CRC increases significantly after the age of 50 and is more common in men. Other factors contributing to the development of this cancer include the presence of hereditary CRC syndromes, such as Lynch syndrome, which increases the risk by 50–80%, and familial adenomatous polyposis. The importance of diet, the negative impact of excess body weight, low physical activity, and smoking on the initiation of colorectal cancer has been demonstrated. Early-stage colorectal cancer (EO-CRC), which occurs in adults under 50, has seen a paradoxical increase in recent years. Patients present with a very advanced stage of the disease at diagnosis, with the tumor being more aggressive than in those typically diagnosed at a later age. Due to its unique molecular and histopathological characteristics, EO-CRC is considered a distinct entity, requiring a tailored approach and separate discussion [4,5].

Several key diagnostic challenges exist in the diagnosis of colorectal cancer, hampering early detection. After a long asymptomatic period, patients with developing CRC may experience symptoms such as abdominal pain, constipation, excessive gas, diarrhea, and changes in stool color and shape. Due to their low specificity and similarity to benign gastrointestinal dysfunction, these symptoms are often underestimated. Consequently, advanced cancer lesions, including metastases, are present at the time of diagnosis, which automatically reduces the effectiveness of treatment initiated at such a late stage [6,7]. Despite the availability of various screening tests, colonoscopy remains the most reliable method for colorectal cancer diagnosis; however, there is still a lack of sensitive molecular biomarkers reflecting tumor biology and immune activation, highlighting the need for cytokine- and chemokine-based approaches [8]. This narrative review aims to summarize current knowledge on cytokine and chemokine signaling networks in colorectal cancer, focusing on their molecular mechanisms, role in tumor microenvironment remodeling, and emerging diagnostic and therapeutic applications.

## 2. Molecular and Immunological Determinants of Colorectal Cancer

**Figure 2:**
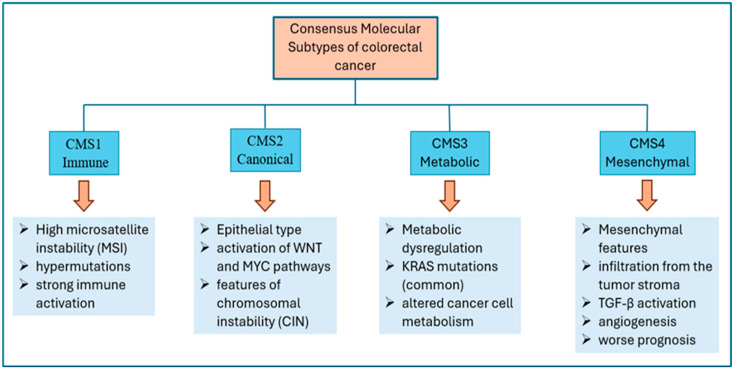
Molecular subtypes of colorectal cancer.

Colorectal cancer is a heterogeneous disease influenced by genetic alterations, the tumor microenvironment, microbiota, and chronic inflammation. In 2014, after conducting a collective analysis of all existing classifications, teams led by Sadanandam and De Sousa E. Melo proposed a new classification of colorectal cancers. As a result of further research and the formation of an international expert consortium, four consensus molecular subtypes (CMS) of colorectal cancer were distinguished: CMS1—immune, CMS2—canonical, CMS3—metabolic, and CMS4—mesenchymal [9] (Figure 2). The canonical subtype is the most common, as it occurs in approximately 37% of cases.

APC mutations cause strong activation of the wingless-type MMTV integration site family (Wnt) signaling pathway and are usually the earliest event in the initiation of colorectal adenomas via the chromosomal instability pathway [10]. Oncogenic KRAS mutations cause constitutive initiation of the epidermal growth factor receptor (EGFR) signaling pathway. CMS3 cancers exhibit metabolic dysregulation of carbohydrates, amino acids, and fatty acids [11]. *Human epidermal growth factor receptor 2* (HER2) gene amplification is found in 7–8% of cases [12]. CMS4 is the second most common subtype of colorectal cancer. It is characterized by concordance with gene signatures of activated stroma, angiogenesis, activation of the transforming growth factor ß (TGF-β) pathway, and proteins associated with microenvironmental inflammation [13]. The immune subtype accounts for approximately 14% of all colorectal cancers, but what distinguishes it from other groups is its distinct biology. Characteristic features include high immunogenicity and microsatellite instability (MSI) due to inactivation of mutator genes (*mutL homolog 1*—MLH1, *mutS homolog 2*—MSH2, *postmeiotic segregation increased 2*—PMS2, and *mutS homolog 6*—MSH6). BRAF V600E hypermutation and mutations in the *phosphatase and tensin homolog* (PTEN) and *ataxia telangiectasia mutated* (ATM) genes can be observed [14]. The canonical CMS2 subtype demonstrates a better survival rate after relapse compared to the other subtypes. Its specific feature is the expression of epithelial signatures via activation of Wnt and MYC proto-oncogene (MYC) signaling [15].

Current literature indicates that, in addition to genetic and environmental risk factors for colorectal cancer, microbiota dysbiosis plays a significant role in its pathogenesis [16]. At least 100 trillion microbial cells inhabit the human body, creating a diverse and incredibly complex ecosystem known as the microbiota. The bacteria, eukaryotes, viruses, and archaea that comprise it constantly interact with each other and with the host [17]. The vast majority of microorganisms reside in the gut microbiota, which plays a crucial role in numerous physiological processes, such as immune system development, immunomodulation, metabolism of dietary compounds, maintaining the structural integrity of the intestinal mucosal barrier, and protection against pathogen invasion and growth. Dysregulation of the intestinal microbiota automatically changes the environment and, consequently, the processes occurring there. This, in turn, disrupts homeostasis in favor of pathological deviations and promotes the development of mutations [17,18].

Many factors, such as inflammation in the body resulting from various processes and disease states, oxidative stress, aging, alcohol abuse, obesity, or chronic infection, lead to damage to the mucosal lining of the colon, resulting in an increased predisposition to the accumulation of mutations within cells and their malignant transformation due to limited immune surveillance [19]. Considering the coexisting inflammation, two subtypes of colorectal cancer can be distinguished: sporadic cancer and colitis-associated cancer (CAC). The former accounts for over 60% of CRC cases and is not associated with variables such as germline mutations or a family history of cancer or inflammatory bowel disease. The latter is closely correlated with long-term inflammation. It is preceded by clinically confirmed inflammatory bowel disease (IBD), with duration and severity of the course being important. A characteristic feature of CAC is the occurrence of polyploid, diffuse, or multifocal, and invasive lesions, unlike sporadic CRC. Literature reports that despite similar frequencies of *PIK3CA*, *BRAF*, and *SMAD4* mutations in both sporadic and CAC, alterations affecting genes responsible for cell motility, cytoskeletal remodeling, and those required for p53 transcription appear more frequently in CAC. Additionally, amplification of the suppressor of cytokine signaling coding region 1 (SOCS1) has been detected in CAC tumors, which downregulates cytokine signaling, including the antitumor activity of interferon gamma (IFN-γ) and interleukin-27 (IL-27) [20]. In both subtypes of CRC, proper communication between the elements of the immune system and the environment is disturbed, which is caused by the release of appropriate cytokines, which, by acting on chemokine networks, additionally fuel and intensify inflammation, leading to destructive effects promoting carcinogenesis [21].

## 3. Inflammation and Tumor Microenvironment in Colorectal Cancer

Scientists’ desire to understand the overall process of carcinogenesis, which, despite enormous research development, new possibilities, and progress, remains incompletely understood, led to the formulation of the cancer immunoediting hypothesis [22]. This hypothesis assumes the inextricable involvement of the immune system in the mechanisms controlling cancer development at every stage. Three phases of the dynamic relationship between cancer cells, leading to tumor development, and the immune system are distinguished: elimination, equilibrium, and escape [22].

The first phase aims to identify cells with any phenotypic changes, such as the presentation of tumor neoantigens or the release of excess damage-associated molecular patterns (DAMPs). They are recognized as dangerous, and their most important function is their elimination [23]. Dendritic cells and macrophages are activated to engulf and present antigens. Natural killer (NK) cells, as a component of the innate immune system, act extremely rapidly because they do not require prior antigen presentation, unlike T lymphocytes. They recognize cancer cells regardless of major histocompatibility complex (MHC) class I expression. They demonstrate direct, effective cytotoxicity through the release of perforins and apoptosis-inducing granzymes and modulate the adaptive immune system. Furthermore, they mediate antibody-dependent cellular cytotoxicity (ADCC) via the Fcγ III receptor [24]. T lymphocytes, including CD8+, CD4+, NKT cells, and γδ T cells, as well as NK cells, secrete key cytokines and effector molecules such as interferon-γ (IFN-γ), tumor necrosis factor (TNF), and TNF-related apoptosis-inducing ligand (TRAIL). B lymphocytes also contribute by differentiating into plasma cells and directly joining the pool of cytokine-secreting cells to most effectively destroy cancer cells [24]. The inflammatory signaling cascade is activated. Secreted interleukin-1 (IL-1) promotes the Th1 response and suppresses Th2. The active form of the potent proinflammatory cytokine IL-1β enhances inflammation by activating native immune cells and producing IL-6 [25].

Cancer cells that survive despite being subjected to intense damaging mechanisms enter the second phase, a state of equilibrium. This can be defined as a period of dormancy, with a permanently sustained suppressive elimination effect [24]. By simultaneously reducing the expression of MHC molecules and costimulatory signals and increasing the expression of immune checkpoint molecules, they evade recognition and inhibit the activation of CD8+ T cells, thus protecting themselves from destruction. Additionally, they can manipulate the immune environment by secreting immunosuppressive cytokines, such as transforming growth factor (TGF-β) and interleukin-10 (IL-10). Their role is to inhibit T cell activation and initiate the differentiation of naive T cells into Treg cells, which are responsible for immunosuppression of the tumor microenvironment [24].

The final stage is the escape phase, which refers to the clinical appearance of the tumor. It is a consequence of genetic instability in rapidly proliferating tumor cells, which have not been detected by components of the immune system [26]. It is a dynamically progressive process, resulting in the appearance of macroscopically identifiable necrotic lesions, which generate and further intensify inflammation and the release of proangiogenic factors that promote further progression. Simultaneously, the function and status of T and NK cells are completely suppressed by the tumor cells, creating a favorable environment for further uncontrolled growth of mutated cells and the development of metastases [24].

Obtaining information regarding the functioning of the tumor microenvironment (TME) has played a significant role in understanding the pathomechanism of cancer. The TME is the area of immediate physical proximity encompassing the tumor stroma, tumor-associated cells, immune cells, endothelial cells, macrophages, vessels, and various components of the extracellular matrix (ECM). The tumor immune microenvironment, composed of the immune components of the TME, primarily myeloid-derived stem cells and regulatory T cells that mediate local immunosuppression, as well as cytotoxic T lymphocytes (CTLs), Th2 cells, and M2 macrophages, is referred to as tumor immune microenvironment (TIME). These components can modulate tumor evolution. Various mediators mediate interactions between all these components, including healthy cells within the tissue and tumor cells with components of the immune system [27].

One type of immune cell belonging to the TME is tumor-associated macrophages (TAMs), which play a significant role in the progression of colorectal cancer. Due to their ability to stimulate signals, mediate epithelial–mesenchymal transition, reprogram lipid metabolism, and interact with fibroblasts that fuse with colonic cells, they are an interesting target worthy of analysis in the context of understanding the mechanisms involved in the processes leading to carcinogenesis [28]. The literature indicates that TAMs primarily originate from circulating inflammatory monocytes, which can be recruited by multiple chemokines, including CCL2 and CCL5, cytokine colony-stimulating factor 1 (CSF1), and their complement cascade [29].

**Figure 3:**
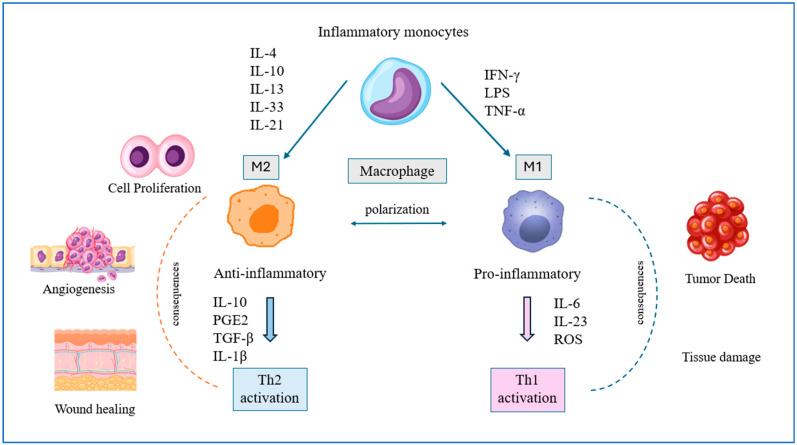
Role of cytokines in macrophage polarization and inflammation-driven tumor progression in colorectal cancer.

As a result of specific differentiation, macrophages can achieve two states of phenotypic polarization (Figure 3). The first is classically activated M1 macrophages with proinflammatory activity. They promote a Th1 response and engulf and subsequently kill target cancer cells. They are induced by IFN-γ, lipopolysaccharide (LPS), or tumor necrosis factor α (TNF-α), among others. By secreting potent inflammatory mediators such as IL-6, interleukin-23 (IL-23), and reactive oxygen species (ROS), they participate in the body’s inflammatory response and, consequently, antitumor immunity. The second type is activated M2 macrophages, which have a counter-inflammatory effect. As a result of induction by cytokines such as IL-4, IL-10, IL-13, IL-33, IL-21, or glucocorticoid-induced differentiation, they increase the production of polyamines and ornithine via the arginase pathway, intensely secrete IL-10, prostaglandin E2 (PGE2), TGF-β, interleukin-1β (IL-1β), and, to a low degree, IL-12. They participate in the Th2 immune response, including humoral and anti-inflammatory immunity. They promote the formation of blood vessel networks, tissue remodeling, and damage repair, while under pathological conditions, they promote tumor initiation, progression, and invasion [30,31]. M2 macrophages present in TAMs silence the antitumor immune response of T lymphocytes, causing abnormal cells to escape control, thus disrupting the interactions between immune system components and their effectiveness. Macrophage activation states are commonly described as above in the M1–M2 spectrum, although it should be noted that in vivo phenotypes are more heterogeneous. The literature indicates the crucial importance of IL-4 and IL-13 as inducers of TAM polarization in the TME [32]. A strong association of tumor-associated macrophages with the expression of the following chemokines, CCL2, CCL3, CCL5, CCL18, CXCL1, and CXCL12, has been demonstrated in the TME. Besides being leukocyte attractants, these molecules can also induce other differentiation mechanisms in monocytes [33]. Cytokine- and chemokine-mediated interactions within the TME create a self-sustaining inflammatory loop that promotes immune evasion and tumor progression in CRC.

## 4. Cytokines in CRC

**Figure 4:**
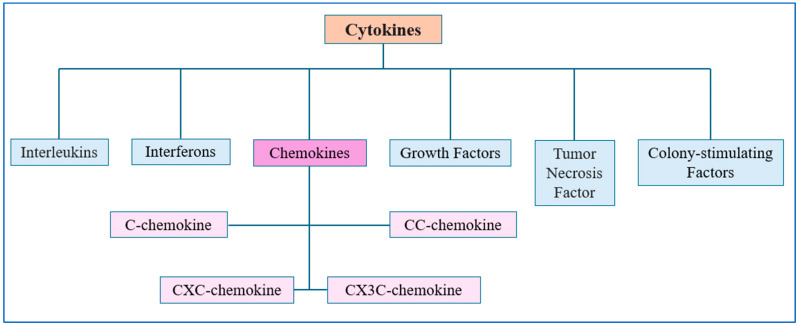
Cytokine classification and chemokine subtypes (C, CC, CXC, CX3C).

Cytokines are soluble proteins involved in the immune response as information mediators and regulators of immune cell maturation, growth, and reactivity. They have a wide range of functions, modulating various processes relevant to the current state of body homeostasis. Cytokines include tumor necrosis factors (TNFs), interleukins (ILs), lymphokines, chemokines, interferons (IFNs), colony-stimulating factors (CSFs), and transforming growth factors (TGFs) (Figure 4). Based on their origin, we distinguish type 1 cytokines, produced by T-helper 1 (T h 1) cluster of differentiation 4 (CD4)+ cells, including IL-2, IL-12, IFN-γ, and TNF-β, and type 2 cytokines, produced by T h 2 CD4+ cells, including IL-4, IL-5, IL-6, IL-10, and IL-13. A clinically important division of cytokines distinguishes those with proinflammatory and anti-inflammatory effects [34]. Cytokine and chemokine profiles vary across CMSs. CMS1 tumors are characterized by high immune infiltration and elevated proinflammatory cytokines, whereas CMS2–4 display more heterogeneous and often immunosuppressed profiles. These differences may influence both disease progression and therapeutic response [15].

### 4.1. Proinflammatory Cytokines

Proinflammatory cytokines are a group of key mediators of the inflammatory response, produced primarily by immune cells such as macrophages, monocytes, and T lymphocytes, but can also be secreted by epithelial cells, fibroblasts, and tumor cells. Their key role is to initiate inflammation, sustain it, and then exacerbate it locally and then systemically. They exert their effects through interactions with specific membrane receptors, activating key signaling pathways such as nuclear factor kappa B (NF-κB), Janus kinase/signal transducer and activator of transcription (JAK/STAT), and mitogen-activated protein kinase (MAPK) [35].

Interleukins are small molecules from the cytokine family that exert pleiotropic effects depending on the phase of health and disease. Their receptors are found on intestinal epithelial cells, but also on tumor cells. They constitute a key element in regulating inflammatory transformation in tumors. On the one hand, by inducing local inflammation, they contribute to the activation of related destructive mechanisms, thus promoting the initiation and subsequent progression of CRC. On the other hand, they are a component of maintaining normal daily intestinal homeostasis by engaging immune system cells in increased activity, secreting additional factors aimed at combating pathogens and destroying mutated cells. There is therefore a fine line between these two opposing mechanisms of action, which, when circumstances change, shift to one side, resulting in corresponding consequences. Proinflammatory cytokines important in the course of colorectal cancer include IL-1β, IL-6, TNF-α, IL-17, and IL-22 [36].

The main source of IL-1β is activated myeloid cells, but it can also be secreted by macrophages, dendritic cells (DCs), monocytes, and neutrophils [37]. Its mechanism of action is complex. Secreted interleukin inactivates glycogen synthase kinase 3β and enhances Wnt signaling, which in turn promotes colon cancer cell growth by creating a self-amplifying loop. This interleukin triggers the overexpression of matrix metalloproteinases (MMPs) in Caco-2 colon cancer epithelial cells by activating protein kinases activator protein 1 (AP-1) and NF-kB [38]. Extracellular IL-1, after binding to the transmembrane receptor IL-1R1, forms a complex with IL-1 receptor accessory protein (IL-1RAP), the adaptor MyD88, and the interleukin 1 receptor-associated kinases IRAK-1, IRAK-2, and IRAK-4. This then leads to a cascade of complex formation and transformation, ultimately generating the transcription factor NF-kB, which enters the cell nucleus and activates transcription of target genes. IL-1β can also induce a signal via MAP kinases and AP-1. It promotes epithelial-to-mesenchymal transition (EMT) and stem cell proliferation in human primary colorectal cancer cells and HCT-116 cells via the ZEB1 protein (Zinc Finger E-box-binding homeobox 1), thus contributing to the progression of colorectal tumors. Through its ability to recruit immunosuppressive TAMs, myeloid-derived suppressor cells (MDSCs), and Tregs, it contributes to angiogenesis, endothelial cell activation, and ultimately, tumor metastasis. A close correlation between IL-1β expression levels and CRC metastasis and, consequently, increased disease progression has been found [39,40,41].

IL-6 is a common cytokine found in the tumor microenvironment. It can be produced by various cells, including tumor-associated macrophages, granulocytes, fibroblasts, and cancer cells. Due to its ability to directly affect cancer cells through binding to the membrane receptor IL-6R (mIL-6R), it induces phosphorylation and activation of the JAK and STAT3 signaling pathways, as well as the expression of target genes [42]. The proteins produced during this process enhance proliferation and facilitate cancer cell survival through modulation of the PI3K/Akt pathway [43,44]. It promotes angiogenesis, invasiveness, metastasis, and general immunosuppression, creating favorable conditions for these events. By activating STAT3, this cytokine increases the expression of the antiapoptotic proteins Bcl-2 and Bcl-xL, while simultaneously suppressing the proapoptotic caspase-3. Furthermore, IL-6 stimulates immune cells to secrete further proinflammatory cytokines, further exacerbating inflammation. IL-6 levels have been shown to correlate with tumor size, stage, and metastasis in patients with colorectal cancer [45]. The immune-rich microenvironment of CMS1 tumors suggests higher responsiveness to immunotherapy, highlighting the importance of considering CMSs when evaluating cytokine signaling and inflammation-related pathways.

Tumor necrosis factor α (TNF-α) is a proinflammatory cytokine produced by hematopoietic and non-hematopoietic cells. Intestinal epithelial cells exhibit high expression of the TNFR1 receptor, which results in increased sensitivity to TNF-α, which plays a key role in the epithelial-to-mesenchymal transition. This interaction intensely activates the NF-kB oncogenic pathway [46]. Its activation increases the expression of early growth factors (EGR1), which participate in promoting CRC cell proliferation and metastasis. However, in colorectal cancer, TNF-α exerts multiple effects and, in addition to pro-tumorigenic processes, may participate in opposing pathways. It can activate the caspase cascade, which induces apoptosis in cancer cells. Furthermore, it activates macrophages and T lymphocytes, which are focused on recognizing and then attacking mutated CRC cells, and recruits other components of the immune system to the tumor site [47].

IL-17 is a proinflammatory cytokine that triggers specific effects upon binding to the IL-17RA receptor. This combination activates mitogen-activated protein kinases (MAPKs), NF-κB, and the CCAAT-enhancer-binding protein (C/EBP) signaling pathways via the adaptor proteins Act1 and TNF receptor-associated factor 6 (TRAF6). The primary source of IL-17 is γδ T cells, but it can also be produced by H17 T cells, innate lymphoid cells (ILC3), natural killer (NK) T cells, or CD8+ cells, which are components of the tumor microenvironment. The nature of its action and the IL-17RA axis on CRC progression is complex and multifaceted, time- and tissue-dependent [48].

IL-22 is a proinflammatory cytokine, elevated levels of which have been detected in various cancers, including stomach, lung, and breast cancers, as well as colorectal cancer. Giannou et al., based on studies conducted in two in vivo mouse models, demonstrated the regulation of liver metastasis progression in CRC by the IL-22-binding protein IL-22BP. The analysis by Baker et al. demonstrated a synergistic relationship between IL-36γ and IL-17α or IL-22, inducing the expression of genes involved in the IL-17/IL-23 axis, which causes the proliferation of colon cancer cells. This suggests the importance of this interleukin in CRC invasion and progression. At the same time, IL-22 may be a useful inflammatory marker enabling early diagnosis and prognostic assessment of colon cancer [49].

### 4.2. Anti-Inflammatory Cytokines

Immunosuppressive cytokines are molecules that control effector antitumor immunity, promoting the survival and proliferation of cancer cells, which consequently enhances cancer growth and progression. These cytokines dominate the tumor microenvironment. The most important anti-inflammatory cytokines supporting tumor development and metastasis in most cancer types, including CRC, include IL-10, transforming growth factor β, IL-4, IL-35, and IL-37 [50].

One of the most important anti-inflammatory cytokines is IL-10. It is produced primarily by immune cells such as regulatory T cells, B cells, macrophages, and dendritic cells. It plays a key role in mitigating inflammation, enhancing the differentiation and immunosuppressive function of Treg cells by promoting STAT3 phosphorylation. It is a potent immunosuppressive factor. By blocking NF-κB signaling through IL-10R on the cell surface, it consequently inhibits the development of proinflammatory cytokines, TGF-1β-driven T cells, and Th17 lymphocytes, which cause colitis [51].

IL-37 is an anti-inflammatory cytokine that suppresses the body’s innate and adaptive immunity. Its function is indirectly due to its ability to inhibit the maturation of dendritic cells and participate in the regulation of macrophage polarization from the M2 to M1 phenotype. It also inhibits the expression and function of proinflammatory cytokines responsible for inflammation, including in the gastrointestinal tract, which is one of the factors predisposing to the development of CRC. Reduced IL-37 levels at both the mRNA and protein levels have been demonstrated in patients with colorectal cancer compared to non-cancerous tissues [52].

Transforming growth factor β (TGF-β) is an important cytokine responsible for maintaining normal cellular homeostasis by inhibiting the cell cycle and inducing apoptosis. However, its high concentration has been shown to correlate with the progression of colorectal cancer. TGF-β is associated with EMT, a reversible biological process that temporarily transforms epithelial cells into a paramesenchymal state. EMT has been found to promote the spread of cancer cells by increasing their mobility, enhancing angiogenesis, matrix remodeling, and stromal infiltration [53,54]. These data suggest that increased TGF-β expression in late-stage colorectal cancer induces a microenvironment favorable to tumor development through direct regulation of both innate and adaptive immune cells and tumor-associated fibroblasts (CAFs). In recent years, this type of fibroblast has attracted particular attention from scientists, who, based on their studies, have identified its importance in the pathogenesis of various types of cancer, including colorectal cancer, recognizing it as a regulator of tumor progression and poorer prognosis. Hawinkels et al. demonstrated a feedback loop between CRC cells and fibroblasts. Accordingly, CAFs are activated by TGF-β, which causes further secretion of this cytokine along with proteinases into the TME, supporting CRC invasion and metastasis [55].

Interleukin-4 (IL-4) is an immunomodulatory and anti-inflammatory cytokine that promotes immune surveillance of cancer. The IL-4 rs2070874 T allele is associated with an overall higher risk of gastrointestinal cancer. This molecule participates in the activation of tumor-associated macrophages and suppressor cells, promoting the development of mutations. Additionally, IL-4 participates in the epithelial–mesenchymal transition in the HCT 116 and RKO colon cancer cell lines via the STAT6 pathway. It promotes macrophage polarization from the M1 to M2 phenotype, which overall facilitates progression and metastasis [56].

IL-35 is a heterodimeric cytokine belonging to the IL-12 family. Compared to IL-10, TGF-β, and IL-4, it was recently discovered in 2007. It consists of the p35 and EBi3 subunits. It induces immunosuppressive iT35 cells from conventional T lymphocytes, responsible for controlling T cell proliferation and CD8 T cell depletion. IL-35 is primarily produced by Treg lymphocytes. Numerous studies, including those by Gu et al., Chen et al., and Fan et al., have demonstrated this effect. Studies have shown its elevated levels in several types of cancer, including prostate, pancreatic, lung, gastric, and colon cancer, which are associated with tumor growth, progression, and clinical stage. This knowledge demonstrates the potential role of IL-35 in modulating the host’s innate and adaptive immune response. Therefore, directly promoting or inhibiting IL-35 and the pathways in which it plays a crucial role may provide therapeutic benefits in cancer treatment [50]. Overall, the balance between pro- and anti-inflammatory cytokines shapes tumor progression and immune evasion, which may vary between CMSs and disease stages.

## 5. Chemokines in CRC

Chemokines are chemotactic proteins from the cytokine family that regulate immune cell migration. They constitute a specific information pathway between cells within tissues and components of the immune system [21]. They are divided into four main subfamilies: CC, CXC, CX3C, and C, distinguished by their basic amino acid sequence and the arrangement of structurally specific cysteine residues linked by disulfide bonds at the N-terminal fragment of the mature protein (Figure 4). By binding to Gαi-protein-coupled seven-transmembrane-spanning receptors (GPCRs) with an N-terminal segment outside the cell and a C-terminal segment with serine and threonine phosphorylation sites in the cytoplasm, they regulate chemotaxis, adhesion, cell positioning, and cell–cell interactions [21,57]. They are molecules involved in both anti- and pro-tumor immune responses, and the ratio of these two functions likely depends on the stage of tumorigenesis, the activation status of immune cells, the balance of effector and regulatory responses, and the expression of chemokine receptors on effector and regulatory target cells. Furthermore, the existence of positive feedback loops dependent on them enhances the predominance of one mechanism over the other, which is directly related to shaping the tumor microenvironment landscape [58]. This suggests that chemokine effects are highly context-dependent, where the same molecule may promote or inhibit tumor progression depending on immune cell composition and tumor stage.

### 5.1. Angiogenic Chemokines

The theory of tumor angiogenesis, first introduced by Judah Folkman in the 1960s, explaining the inextricable link between the formation of a dense network of blood vessels and tumor development, has, over the years, only strengthened scientists’ conviction of its validity through numerous studies and observations [59]. Today, it is known that tumor cells, with their rapid rate of division and differentiation, require large amounts of oxygen and nutrients, resulting in the dynamic formation of new blood vessels. This process is regulated by pro- and antiangiogenic substances secreted by tumor cells, playing a key role in tumor growth, invasion, and metastasis [60]. In hypoxic conditions, angiogenic factors bind to a receptor on the surface of endothelial cells, causing their dilation and activation. This leads to increased expression of certain proteases, initiating basement membrane degradation and pericyte detachment. Highly mobile apical endothelial cells migrate upon exposure to an angiogenic stimulus. This leads to cell proliferation, inducing the formation of new blood vessels, which then coalesce to form a network that supplies the tumor, thereby stimulating its growth and metastasis, which significantly worsens the prognosis [61,62].

The most important inducer of tumor angiogenesis is the vascular endothelial growth factor (VEGF) family, consisting of five proteins: VEGF-A, VEGF-B, VEGF-C, VEGF-D, and placental growth factor (PlGF). VEGF-A, originally known as vascular permeability factor (VPF), has the strongest effect [61,63]. Their biological function is achieved through interaction with the following receptors: VEGFR-1, VEGFR-2, VEGFR-3, and two co-receptors: neuropilin and heparan sulfate proteoglycans. While VEGF-B, PlGF, and VEGF-A bind to VEGFR-1, VEGF-A, VEGF-C, and VEGF-D bind to VEGFR-2, VEGF-C and VEGF-D bind to VEGFR-3 [61]. Other initiators of tumor angiogenesis include fibroblast growth factor-2 (FGF2), the platelet-derived growth factor (PDGF) family, angiopoietins, Eph proteins, and apelin (APLN) [63].

Chemokines play an equally important role in tumor angiogenesis, either directly binding to receptors on endothelial cells or indirectly through the recruitment of inflammatory cells and progenitors. ELR + CXC motif (CXC) chemokines, including CXCL1, CXCL2, CXCL3, CXCL5, CXCL6, CXCL7, and CXCL8, induce the formation of new blood vessels by binding to the common receptor CXCR2, thereby activating the AKT/NF-κB/FOXD1/VEGF-A pathway. VEGF activity also induces increased expression of CXCL1 and CXCL8 in endothelial cells, which further enhances the angiogenesis process and thus contributes to tumor development. Each of these molecules exerts its functions, directly or indirectly, affecting tumor cells, constituting a crucial link in the processes occurring within the tumor microenvironment. Below, we will focus on a more detailed characterization of several selected proangiogenic chemokines that have attracted particular attention as potentially useful in the diagnosis and prognosis of patients with colorectal cancer [63,64].

CXCL8/IL-8 is a proangiogenic, proinflammatory CXC ELR (Glu-Leu-Arg)+ chemokine that exerts its effects by binding to its cognate receptors (CXC receptor (CXCR) 1 and 2), two G protein-coupled receptors expressed by both immune, stromal, and tumor cells. This initiates a phosphoinositide 3-kinase [PI3K] and mitogen-activated protein kinase [MAPK] cascade. Downstream PI3K and MAPK signaling promote protein translation as well as tumor cell proliferation and survival. As a result of binding to the CXCR1 and CXCR2 receptors expressed by endothelial cells, CXCL8 leads to increased endothelial cell division and capillary reorganization, which in turn contributes to epithelial–mesenchymal transition (EMT) and the generation and maintenance of cancer stem cells (CSCs) in the colon. CXCL8/IL-8 is also a major chemoattractant of monocytes/macrophages in cancer tissue, and when secreted by tumor-associated macrophages, it predisposes to metastasis. Interestingly, CRC cells can autocrinally regulate CXCL8 and increase CXCR1 and CXCR2 expression, which further contributes to the spread of metastases throughout the body [65]. Scientists have confirmed that CXCL8 levels in blood and tissues are strongly associated with treatment outcomes and have prognostic significance. Some clinical studies have reported reduced relapse-free survival and overall survival in colorectal cancer patients with high CXCL8 expression on epithelial cells. Accordingly, elevated serum concentrations of this chemokine have been observed in CRC patients, varying depending on the stage of the disease compared to healthy individuals, confirming the correlation between its levels and the stage of CRC progression [66].

CCL2, also known as monocyte chemotactic protein-1 (MCP-1), was the first CC chemokine discovered and studied in 1989. It is produced by many cell types, including cancer cells, endothelial cells, fibroblasts, epithelial cells, smooth muscle cells, and myeloid cells. It exerts its biological effects by binding to the CCR2 receptor. Activation of CCR2 by its ligand CCL2 activates the phosphatidylinositol 3-kinase (PI3K)/AKT, mitogen-activated protein kinase (MAPK)/p38, and Janus kinase (JAK)/STAT3 cascades. Their activation is crucial in inhibiting apoptosis, angiogenesis, and cell migration, leading to the development of oncogenes. The CCL2-CCR2 signaling axis is important in the occurrence and progression of a wide range of malignancies, including breast, prostate, pancreas, and colon cancers. CCL2-CCR2 can stimulate cancer cells to invade surrounding tissues, enter the circulatory system, and spread along a specific chemotactic gradient to metastatic sites. CCL2 recruits CCR2+ TAMs, MDSCs, and Th2 cells, thus creating an immunosuppressive microenvironment that contributes to tumor progression [67].

The chemokine CXCL12, also known as stromal-derived factor 1 (SDF-1), is an extracellular, proangiogenic chemokine that binds to the CXCR4 (CD184) and CXCR7 receptors [68]. Its primary function is to regulate hematopoietic cell trafficking and the architecture of secondary lymphoid tissues. Despite being ELR-negative, it is one of the most potent chemokines promoting angiogenesis through activation of key survival pathways regulated by ERK/MAPK, Jak/STAT, and PI3K/Akt/mTOR signaling. CXCL12 facilitates tumor dissemination, influencing endothelial adhesion, vascular eruption, proliferation, angiogenesis, colonization, and evasion of the host response [69]. It interacts with integrins essential for cell adhesion and recruits tumor-associated neutrophils and macrophages, contributing to shaping the tumor microenvironment. Increased expression and role of the CXCL12–CXCR4 axis have been studied and observed in tumors of the kidney, lung, brain, prostate, colon, breast, pancreas, ovary, and endometrium. During rapid tumor progression, leading to hypoxia in the TME, increased production of CXCR4 and CXCL12 occurs in monocytes, monocyte-derived macrophages, tumor-associated macrophages, endothelial cells, and cancer cells. Researchers have demonstrated that CXCL12-CXCR4 is involved in metastasis in CRC, melanoma, and pancreas, among others. Furthermore, increased CXCL12 expression in hepatocytes is associated with increased levels of CXCR4 (+) cells in melanoma and CRC liver metastases via Wnt/β-catenin signaling. Furthermore, the research team of Xu H et al. identified a potential role for CXCR7 in the development, growth, and metastasis of CRC via TLR4 signaling. CXCR7 gene silencing leads to cell apoptosis and inhibition of CRC via the ERK1/2 and β-arrestin2 pathways, regulating the expression of MMP-2 and caspase-3 [68].

### 5.2. Angiostatic Chemokines

The second group of chemokines is those with anti-angiogenic activity. These signaling proteins are crucial in calming inflammation and inhibiting tumor growth by blocking the development of the vascular system. However, their expression levels are crucial and predispose them to play various roles in other pathways, even pro-tumor ones. Examples of antiangiogenic chemokines include CXCL4, CXCL9, CXCL10, CXCL11, and CXCL13 [70]. The effects of CXCL13 appear to be context-dependent. It has been reported to exhibit angiostatic properties while also contributing to immunosuppression by recruiting myeloid-derived suppressor cells [70].

CXCL4, also known as C–X–C chemokine motif ligand 4 or platelet factor 4 (PF4), is an ELR-negative chemokine with two sources. CXCL4, derived from platelets, is stored within them and released in large quantities upon activation, thereby regulating blood coagulation. The second type of CXCL4, not derived from platelets, is produced and secreted by some somatic cells, such as lymphocytes. It acts through the chemokine receptor CXCR3, expressed on various cell types, including cancer cells. CXCL4 has been shown to induce the production of MDSCs, which negatively regulate CD8+ T cell function [71]. PF4 inhibits the differentiation of Th17 lymphocytes into T helper lymphocytes, reduces IFN-γ levels in Th1 cells, and increases IL-4, IL-5, and IL-13 levels in Th2 cells at the mRNA level [72]. Through direct association with fibroblast growth factor 2 (FGF-2), it inhibits the dimerization of the molecule and blocks the binding of FGF-2 to endothelial cells, which consequently leads to inhibition of angiogenesis. Additionally, CXCL4 inhibits endothelial cell proliferation and migration by inhibiting VEGF165 binding to VEGFR-2 and blocking VEGF-121. It induces apoptosis, while simultaneously antagonizing the effects of ELR+ factors [73,74].

CXCL9 is a member of the CXC chemokine family that plays a key role in modulating antitumor immunity. Literature indicates a significant correlation between this molecule and other chemokines within the CXC family, namely CXCL10, CXCL11, and CXCL13. These proteins can attract immune cells such as T lymphocytes, natural killer (NK) cells, and monocytes to sites of inflammation or infection, as well as to the tumor microenvironment. CXCL9 may play a dual role in regulating mechanisms in the TME, both promoting and inhibiting tumor progression, depending on the context. On the one hand, studies conducted by scientists have shown elevated levels of CXCL9 in the tissues of patients with colorectal cancer, which correlates with advanced disease stages and tumor proliferation, invasion, metastasis, and autophagy. On the other hand, by modulating the body’s immune response in the tumor microenvironment, it plays a protective role. Furthermore, high CXCL9 expression partially mitigates the tumor-suppressive effects observed after ALKBH5 downregulation and is associated with improved overall survival and a better response to immunotherapy in patients with CRC [75].

CXCL10 is a chemokine from the CXC family, crucial for the recruitment of immune cells and the modulation of inflammation, particularly in the tumor microenvironment. Its expression occurs primarily in macrophages and is modulated by interferon-γ (IFN-γ) and tumor necrosis factor alpha (TNF-α). Its biological effects are mediated by binding to the CXCR3 receptor, which is highly expressed on CD8+ T cells, NK cells, and Th1 CD4+ T cells. The resulting CXCL10-CXCR3 signaling axis activates several intracellular signaling pathways, such as JAK/STAT, MAPK/ERK, and PI3K/Akt, which modulate and coordinate the inflammatory process, immune cell response, and other components within the tumor microenvironment. It promotes macrophage polarization from M1 to M2, thereby supporting the production of proinflammatory cytokines and reactive oxygen species. All of these complex processes, in which CXCL10 is a link, interacting with each other at various stages, shape the entire tumor immune system, determining the intensity and nature of antitumor immunity. Although CXCL10 controls the effective response of the body against tumors, including those of the colon, it can paradoxically be used by tumors in certain situations to promote immune evasion and CRC progression [76,77].

## 6. Diagnostic Potential of Cytokines and Chemokines

The development of CRC is a multi-stage, long process involving numerous histological, morphological, and genetic abnormalities. One in four patients with colorectal cancer already has metastases at the time of diagnosis. These most often involve the liver and significantly worsen the prognosis. Therefore, methods enabling the earliest detection of lesions are extremely important [78]. In addition to screening and imaging tests, laboratory diagnostics play a crucial role. Its greatest advantages include non-invasiveness, easy availability, no need for patient preparation, and a short turnaround time. Laboratory tests include the determination of classic serum markers such as carcinoembryonic antigen (CEA) and CA-19-9. These tests support the monitoring of patients already diagnosed during treatment and enable the detection of recurrence. However, they do not have diagnostic capabilities; hence, attempts have been made to find and study new molecules that could serve as diagnostic biomarkers, i.e., those whose appearance or change in concentration in blood, urine, feces, tissues, or other body fluids indicates the presence of cancer in the patient [79]. Tissue and serum biomarkers have undoubted potential. The advantage of tissue biomarkers is their high expression due to their local involvement in processes occurring within the tumor microenvironment, while serum biomarkers are more readily available and do not require biopsy. Changes in their concentrations reflect a broader, systemic state in the body, more closely related to disease progression. However, the frequent correlation between concentration changes in both types of biological material is emphasized. Biomarkers can be various types of circulating biochemical molecules, such as proteins, tumor DNA, tumor-derived cells, and miRNA [80]. Cytokines and chemokines are currently of great interest among scientists due to the fact that they participate in many mechanisms, including processes initiating the development and proliferation of cancer cells in the large intestine. Despite the availability of ctDNA-based assays that provide information on tumor-derived genetic alterations and minimal residual disease, circulating cytokines and chemokines reflect the dynamic interaction between the tumor and the host immune system, which adds value [63,64].

Most proinflammatory cytokines, such as IL-6, IL-17A, and TNF-α, promote tumor growth by stimulating cell proliferation, angiogenesis, and remodeling the tumor microenvironment. Some cytokines, including IL-2, IL-12, and IL-15, exert antitumor effects by activating cytotoxic cells and enhancing the immune response. Dual-role cytokines (IL-18, TNF-α) can both promote tumor growth and trigger antitumor mechanisms, highlighting the complexity of their role in colorectal cancer (Table 1).

Angiogenic chemokines, such as CXCL1, CXCL8, and CCL2, accelerate tumor progression by recruiting immunosuppressive cells, promoting proliferation and metastasis. In contrast, angiostatic chemokines, such as CXCL9–CXCL11 and CXCL4, exert antitumor effects by recruiting cytotoxic lymphocytes to the tumor microenvironment. Therefore, chemokines play a crucial role in both colorectal cancer progression and immune surveillance, making them potential diagnostic biomarkers and therapeutic targets (Table 2). Importantly, the biological and clinical significance of individual cytokines and chemokines in colorectal cancer varies considerably depending on tumor stage, molecular subtype, cellular source, and composition of the tumor microenvironment. These differences limit direct comparison of studies and currently hinder their translation into routine clinical biomarkers. These observations underscore the potential for CMS-stratified biomarker selection and tailored therapeutic strategies, particularly by leveraging the immunogenic features of CMS1 tumors.

The selected cytokines and chemokines discussed here, as molecules predisposing to the role of biomarkers in colorectal cancer, may complement the existing panel of laboratory parameters, enhancing their diagnostic and prognostic value (Table 3). An example of this is the study by Xiao J et al., who demonstrated improved diagnostic accuracy when CEA was measured concurrently with IL-6, compared to carcinoembryonic antigen alone, in patients with colorectal cancer [88].

As with any candidate biomarker, cytokines and chemokines unfortunately have their limitations. Although research shows significant changes in their concentrations in patients with colon cancer compared to healthy individuals, other publications report changes in their levels also in the course of cancers affecting other organs, but not exclusively. The presence of inflammation, both acute and chronic, is crucial. It accompanies many diseases, such as infections, trauma, metabolic diseases, and autoimmune diseases such as lupus, multiple sclerosis, and rheumatoid arthritis. In this case, it is crucial to exclude them in order to accurately interpret the patient’s clinical situation related to tumorigenesis in the colon [35,89,90].

**Table 3:** Comparison of diagnostic indicators of selected cytokines/chemokines in colorectal cancer.

Cytokine/Chemokine	Diagnostic Sensitivity	Diagnostic Specificity	AUC	Ref.
IL-2	~73.3%	~64.9%	~0.706	[91]
IL-6	~60.6–72.2%	~74.0–83.9%	0.75–0.82	[92]
CXCL5	~57–68%	~58–67%	0.65	[93,94]
CXCL8	~69–86.34%	54.05–81.3	0.742–0.920	[95,96]
CCL2	~64%	~60%	~0.634	[97]
TNF-α	~53–96%	~54–87%	0.88	[98]

AUC—area under the ROC curve.

## 7. Therapeutic Applications of Cytokines and Chemokines

The traditional pillars of the classic therapeutic strategy for colorectal cancer are surgery, chemotherapy, and targeted therapy. Surgery is most effective in patients with early-stage disease, i.e., with lesions limited to the colon. However, approximately 20% of cases already have distant metastases at diagnosis, automatically ruling this method out in favor of chemotherapy [99]. Classic chemotherapy, directed at rapidly dividing cells, aims to disrupt DNA replication, mitosis, and metabolic pathways essential for cell proliferation. For years, this type of treatment focused on various combinations of 5-fluorouracil (5FU), irinotecan, oxaliplatin, and leucovorin. Due to several side effects, primarily the negative impact on healthy cells in the bone marrow and gastrointestinal tract, a new therapeutic approach was needed [100].

Despite strenuous attempts to optimize the therapeutic process using the above-mentioned method, the results were often limited, leading to a reorganization of colorectal cancer treatment [99]. Unlike classical chemotherapy, targeted therapy is designed to act directly on cancer cells, blocking molecular pathways and all processes that enable their proliferation [100,101]. This strategy is based on the use of monoclonal antibodies (mAbs) and small-molecule inhibitors. In patients with colorectal cancer, mAbs such as cetuximab and panitumumab, which have the ability to block the epidermal growth factor receptor, are used. Both antibodies have been shown to reduce the likelihood of tumor progression and improve clinical outcomes in selected patient, improving overall survival, progression-free survival, and quality of life in patients with refractory CRC. Bevacizumab and ramucirumab, on the other hand, are antibodies that block vascular endothelial growth factor (VEGF), thereby reducing blood flow to the tumor, depriving it of nutrients and oxygen, as reported in the randomized phase II PARERE trial [102].

Programmed cell death protein 1 (PD-1) and its ligand, PD-L1, have become important therapeutic targets in immunotherapy, a method that involves engaging the patient’s immune system in the fight against the tumor. Monoclonal antibodies targeting PD-1/PD-L1 have been used in patients with CRC and have shown a survival benefit, including CRC cases with high microsatellite instability (MSI-H) and mismatch repair deficiency (dMMR). PD-1 (CD279) is a protein that acts as a co-inhibitory receptor, inhibiting excessive immune activation. By binding to PD-L1 (CD274) and PD-L2 ligands, which are present on tumor cells and immune cells in the tumor microenvironment (TME), it attenuates T-cell receptor (TCR) signaling and costimulatory pathways, promoting immune escape. Expression of this protein in tumors is regulated by inflammatory cytokines such as interferon gamma (IFN-γ), hypoxia-inducible factors, genomic alterations, and epigenetic modifications. Pembrolizumab, nivolumab, and dostarlimab are PD-1/PD-L1 inhibitors that block the immune checkpoint interaction between PD-1 on T cells and PD-L1 on tumor cells, restoring T-cell activity and enabling an antitumor immune response. Clinical trials such as phase 2 study, CheckMate 142 indicate the effectiveness of this type of therapy in patients with MSI-H/dMMR colorectal cancer. However, the problem is that most CRC cases are characterized by microsatellite stability (MSS), highlighting the need for alternative or complementary therapeutic strategies [103]. Another approach included the CCR5 inhibitor maraviroc in combination with pembrolizumab, which resulted in increased levels of anticancer chemokines, as demonstrated by the Phase I PICCASSO study. Another proposal by scientists is the colony-stimulating factor receptor 1 inhibitor AMG 820, and NOX-A12, a CXCL12 inhibitor, which are being explored based on their potential to modulate the TME and enhance the immune response through increased levels of inflammatory cytokines and a greater number of activated CD3+ T cells [104]. These approaches suggest potential therapeutic avenues, although further validation is required. It is also important to note that therapeutic efficacy and safety profiles can vary considerably between studies. This reflects differences in patient populations, molecular subtypes of CRC and treatment protocols. These results require confirmation in well-designed, prospective trials.

A complete understanding of the functions and mechanisms involved in individual cytokines and chemokines creates the conditions for effective, and above all, highly efficient, treatment. The greatest challenge is applying the right cytokine/chemokine to the appropriate site to maximize their therapeutic support. The route of administration is also crucial, as systemic administration of cytokines results in moderate to severe side effects, such as fever, chills, fatigue, nausea, depression, and changes in blood counts. This automatically reduces clinical utility, while the presence of toxic effects disqualifies a given compound despite its potential [105].

With their knowledge of the action and role of IL-2 and IL-12 in anticancer processes, scientists decided to investigate whether these molecules could be used in immunotherapy. Studies involving, among others, the delivery of modified IL-12 directly to tumor tissues using viral, non-viral, and cellular vectors, fusion of IL-12 with extracellular matrix proteins, collagen, and immunological factors, enable the achievement of high concentrations within the tumor that suggests therapeutic potential. Additional interest has been generated by the binding of IL-12 to immunological factors such as the human tandem antibody L19, IL-12 derivatives combining a fragment of the anti-EDB antibody scFv(L19), and NHS-muIL12-combining antibody with a DNA/DNA-histone complex, which may enhance antitumor activity by increasing production of IFN-γ and activating NK cells and CD8+ T lymphocytes [106]. Due to IL-2’s ability to activate and expand cytotoxic effector cells, it has been studied in numerous clinical trials over the years, and in 1992, it was approved by the US Food and Drug Administration (US-FDA) as high-dose (HD) IL-2 (aldesleukin) for the treatment of renal cell carcinoma (RCC). Despite promising results, its use has currently been limited due to toxic side effects in both responding and non-responding patients [107]. Moreover, the available evidence is largely derived from early-phase or exploratory studies, which exhibit substantial variability in terms of study design, patient selection and outcome measures.

Clinical trials have been conducted using monotherapy with drugs targeting cytokine signaling, aiming to inhibit or stimulate it. An example is the IL-1 antagonist anakinra, which has been studied to minimize the proinflammatory and pro-tumorigenic properties of IL-1 in CRC. However, due to cytokine redundancy and pathway crosstalk, cytokine-targeted monotherapy has shown limited efficacy, highlighting the need for combination strategies [20]. Combining cytokine-modulation therapy with various anticancer drugs and protocols is preferable. An example is NCAGN01876, an antibody directed against GITR, a member of the TNF receptor family, in combination with nivolumab and ipilimumab. The goal was to increase the efficacy of anti-PD1 and anti-CTLA-4 therapy in colorectal cancer, or IFN-γ in combination with 5-fluorouracil, leucovorin, and with or without bevacizumab, to assess safety, tolerability, and efficacy [20].

Many types of cancer, including colorectal cancer, experience overexpression of CXCR4, which, through the CXCR4/CXC12 axis, has been consistently associated with tumor progression, making it an attractive target for diagnosis and therapy. Blocking this axis by binding the CXCR4 receptor to appropriate ligands, either agonists or antagonists, has therefore been actively investigated. This has unique therapeutic potential in cancer treatment and/or targeted drug delivery. An example of a CXCR4 receptor agonist is CTCE-0214, a modified version of the SDF-1α peptide. Its role is to promote tissue repair and immune cell migration. ATI-2341, also known as pepducin, modulates cellular responses by promoting biased signaling between G proteins and β-arrestins. Extensive research which has reached phase 1 clinical trials (NCT02179970) has demonstrated the lower stability and benefit of CXCR4 receptor agonists compared to antagonists, which hold greater therapeutic promise. In turn, ATI-2341, also known as pepducin, modulates cellular responses by promoting biased signaling between G proteins and β-arrestins. Extensive research has demonstrated the lower stability and benefit of CXCR4 receptor agonists compared to antagonists, which hold greater therapeutic promise. Antagonists include numerous synthetic and natural peptides, such as [Tyr5,12,Lys7]-polyphemusin II, CVX15, LY2510924, and CTCE-9908. In addition, monoclonal antibodies include Ulocuplumab, which is also known as BMS-936564 or MDX-1338, or PF-06747143—a humanized IgG1 anti-CXCR4 antibody from Pfizer, and small molecules, e.g., AMD3100—a bicyclam derivative, and natural products such as penicillisanthone A (PXA), a flavonoid dimer of marine origin, acting as a CCR5/CXCR4 receptor antagonist, or sacosaponin A [108]. Although classical surgery and chemotherapy remain the mainstays of treatment for colorectal cancer, advances in targeted therapy, immunotherapy, and the use of cytokines and chemokines represent promising avenues that require further clinical validation, with the central challenge being the balance between efficacy and toxicity, particularly when modulating the immune response and tumor microenvironment.

## 8. Conclusions and Future Perspectives

The search for new molecules, compounds, and therapeutic combinations is underway to enable earlier detection of the first cancerous lesions at an early stage, inhibit their progression, and potentially lead to effective treatment for patients, whose number is increasing year by year, as indicated by global incidence statistics. Therefore, biomarkers are considered a key tool for early detection, prognosis, and predicting treatment response, as well as cancer patients’ survival. It is essential to develop new biochemical markers that are widely available, easy to measure, inexpensive, and, above all, exhibit high sensitivity and specificity.

The most frequently reported cytokines with the potential to be incorporated into routine diagnostics include IL-6, CXCL-8, and CCL2. Numerous studies have demonstrated associations between their levels and the stage of CRC advancement, and have linked elevated concentrations to poorer prognosis due to their proinflammatory and proangiogenic effects, their ability to migrate, and their ability to recruit immunosuppressive cells. Achieving the highest possible accuracy and specificity, which translates into usefulness, is suggested to be more likely by using a panel approach, i.e., simultaneously measuring several markers. Furthermore, the use of CXCL12–CXCR4 in the context of liver metastases, which are the most common site of distant metastases, has been associated with advanced disease progression and has attracted considerable research interest. Numerous clinical trials have investigated the potential use CXCR4/CXC12 axis inhibitors, PD-1/PD-L1 antibodies, or directly IL-2 or IL-12. Despite their proposed effectiveness in blocking cancer cell proliferation, the greatest challenge lies in determining the appropriate route and site of administration, a therapeutic, non-toxic dose, and a treatment duration that is free from adverse effects. Moreover, these findings are derived from heterogeneous patient cohorts and employ diverse analytical platforms and assay protocols. They also largely lack prospective validation, which currently limits their routine clinical implementation [20,100,101,102,103,104,106,108].

In summary, the selected cytokines and chemokines discussed in the above review are involved in multiple processes that contribute to the development and progression of colorectal cancer. By recruiting appropriate immune cells, they modulate the tumor microenvironment, thus influencing its growth, progression, and metastasis. Clinical studies have reported that cytokine and chemokine levels correlate with tumor stage, highlighting their potential both as diagnostic biomarkers and as targets for novel therapies. However, while this direction is considered promising, further research and prospective validation are required before these approaches can be implemented in routine clinical practice.

## Figures and Tables

**Table 1:** Cytokines in colorectal cancer.

Cytokine	Effect	Key Mechanism	References
IL-1	Proinflammatory	Activates NF-κB, JNK, AP-1, p38 MAPK; Wnt signaling via GSK3β	[56,81]
IL-1β	Proinflammatory	Activates myeloid cells, enhances IL-17A response, promotes CRC proliferation and microenvironment changes, induces TNF-α, IL-6, IL-8, COX-2, PGE2	[56,81]
IL-2	Proinflammatory	Stimulates T, B, NK cells; increases CD4/CD8 differentiation; mediates Treg development; anticancer activity	[57,82]
IL-6	Proinflammatory	Promotes proliferation, metastasis (IL-6/JAK/STAT3), angiogenesis; inhibits apoptosis; suppresses Th1 differentiation; slows DC maturation	[81,83]
IL-8 (CXCL8)	Proinflammatory	Promotes tumor growth and metastasis primarily through angiogenesis, neutrophil recruitment, and activation of pro-tumorigenic signaling pathways	[84,85]
IL-10	Predominantly anti-inflammatory (context-dependent effects)	Predominantly immunosuppressive; reduces IL-2 and IFN-γ production; stimulates B cells; inhibits TAA cross-presentation; may enhance CD8^+^ T-cell cytotoxicity in specific experimental contexts	[50,51]
IL-12	Proinflammatory	Activates NK/T cells; phosphorylates STAT1–5; suppresses VEGF via IFNγ	[86]
IL-15	Proinflammatory	Inhibits CRC; promotes T/NK cell activation and proliferation	[81,82]
IL-17A	Proinflammatory	Supports tumor growth via IL-6/STAT3; promotes angiogenesis (VEGF, PGE2); cell cycle progression	[81,82,85]
IL-18	Dual	Antitumor: activates cytotoxic T/NK cells; Protumor: promotes angiogenesis/metastasis, immune evasion	[82]
IL-21	Proinflammatory	Activates immune response biomarkers; increases CD8, NK, NKT cytotoxicity	[82]
IL-22	Proinflammatory	Promotes tumor growth via STAT3/DOT1L signaling	[56]
IL-33	Proinflammatory	Facilitates tumor immune escape via Th2/Treg cells	[85,87]
TNF-α	Proinflammatory	Pro-tumor: NF-κB/STAT3 activation, angiogenesis; Anti-tumor: increases tumor vessel permeability, supports chemo	[57,82]

**Table 2:** Chemokines in colorectal cancer.

Chemokine	Receptor	Effect on Angiogenesis	Clinical Significance	References
CXCL1	CXCR2	Angiogenic	Recruits neutrophils and MDSCs; promotes proliferation; tumor budding; increases glycolysis and PTHLH	[63,64]
CXCL2	CXCR2	Angiogenic	Recruits neutrophils and MDSCs	[60,61]
CXCL3	CXCR2	Angiogenic	–	[60]
CXCL4	CXCR3	Angiostatic	Anti-tumor via TIL recruitment	[70,71]
CXCL5	CXCR2	Angiogenic	Recruits neutrophils and MDSCs	[60,62]
CXCL6	CXCR1/CXCR2	Angiogenic	–	[60]
CXCL7	CXCR2	Angiogenic	Recruits TAMs	[60,61]
CXCL8	CXCR1/CXCR2	Angiogenic	Recruits neutrophils, macrophages and MDSCs	[64,76]
CXCL9	CXCR3	Angiostatic	Anti-tumor via TIL recruitment	[70,75]
CXCL10/11	CXCR3	Angiostatic	Anti-tumor via TIL recruitment	[76,77]
CXCL12	CXCR4/CXCR7	Angiogenic/ Lymphangiogenic	Recruits neutrophils, macrophages, MDSCs; regulates integrins, ERK/MAPK, JAK/STAT, PI3K/Akt/mTOR; promotes metastasis	[68,69]
CXCL13	CXCR3/CXCR5	Angiostatic (context-dependent)	Recruits MDSCs (immunosuppressive context)	[70]
CXCL16	CXCR6/ mCXCL16	Angiogenic	Recruits TAMs; anti-tumor via TILs	[73]
CXCL17	CXCR8	Angiogenic	Recruits MDSCs	[73]
CCL2	CCR2	Angiogenic	Attracts monocytes, stimulates M2 macrophages; increases endothelial permeability; promotes tumor progression	[67]
CCL5	CCR5	Angiogenic	Enhances migration, invasion, and survival; suppresses immune response	[58]

Note: Chemokine receptor usage may vary depending on cellular context, receptor isoforms, and experimental conditions. Some ligands may signal through alternative receptors in specific tumor microenvironment settings.

## Data Availability

No new data were created or analyzed in this study. Data sharing is not applicable to this article.
